# Genome-wide association study and trans-ethnic meta-analysis identify novel susceptibility loci for type 2 diabetes mellitus

**DOI:** 10.1186/s12920-024-01855-1

**Published:** 2024-04-29

**Authors:** Asma A Elashi, Salman M Toor, Umm-Kulthum Ismail Umlai, Yasser A Al-Sarraj, Shahrad Taheri, Karsten Suhre, Abdul Badi Abou-Samra, Omar M E Albagha

**Affiliations:** 1 College of Health and Life Sciences (CHLS), Hamad Bin Khalifa University (HBKU), Qatar Foundation (QF), Education City, Doha, P.O. Box 34110, Qatar; 2 Qatar Genome Program (QGP), Qatar Foundation Research, Development and Innovation, Qatar Foundation (QF), Doha, P.O. Box 5825, Qatar; 3 Qatar Metabolic Institute, Hamad Medical Corporation, P.O. Box 3050 Doha, Qatar; 4 Bioinformatics Core, Weill Cornell Medicine-Qatar, Education City, Doha, P.O. Box 24144, Qatar; 5 Department of Biophysics and Physiology, Weill Cornell Medicine, 510065 New York, USA; 6 Centre for Genomic and Experimental Medicine, Institute of Genetics and Cancer, University of Edinburgh, EH4 2XU Edinburgh, UK

**Keywords:** GWAS, Type 2 diabetes Mellitus, T2D, SNP, *TCF7L2*, *DYNC2H1*, Polygenic risk score

## Abstract

**Background:**

The genetic basis of type 2 diabetes (T2D) is under-investigated in the Middle East, despite the rapidly growing disease prevalence. We aimed to define the genetic determinants of T2D in Qatar.

**Methods:**

Using whole genome sequencing of 11,436 participants (2765 T2D cases and 8671 controls) from the population-based Qatar Biobank (QBB), we conducted a genome-wide association study (GWAS) of T2D with and without body mass index (BMI) adjustment.

**Results:**

We replicated 93 known T2D-associated loci in a BMI-unadjusted model, while 96 known loci were replicated in a BMI-adjusted model. The effect sizes and allele frequencies of replicated SNPs in the Qatari population generally concurred with those from European populations. We identified a locus specific to our cohort located between the *APOBEC3H* and *CBX7* genes in the BMI-unadjusted model. Also, we performed a transethnic meta-analysis of our cohort with a previous GWAS on T2D in multi-ancestry individuals (180,834 T2D cases and 1,159,055 controls). One locus in *DYNC2H1* gene reached genome-wide significance in the meta-analysis. Assessing polygenic risk scores derived from European- and multi-ancestries in the Qatari population showed higher predictive performance of the multi-ancestry panel compared to the European panel.

**Conclusion:**

Our study provides new insights into the genetic architecture of T2D in a Middle Eastern population and identifies genes that may be explored further for their involvement in T2D pathogenesis.

**Supplementary Information:**

The online version contains supplementary material available at 10.1186/s12920-024-01855-1.

## Background

The prevalence of diabetes in the Middle East and North Africa (MENA) region is amongst the highest in the world at ∼ 16% and accounts for ∼ 18% of the world’s overall adult diabetic population of more than 500 million individuals in 2021 [1]. Diabetes currently affects ∼ 1 in 6 adults in the MENA region and is predicted to affect ∼ 1 in 5 adults, equating to ∼ 136 million individuals with diabetes by 2045 [1]. Type 2 diabetes (T2D) accounts for ∼ 90% of all diabetes cases and has a strong genetic predisposition [2]. T2D is a chronic disease associated with high morbidity and mortality. Individuals with T2D exhibit macrovascular complications, including cardiovascular disease, which is the leading cause of death in T2D [3]. Moreover, around one-third of patients with T2D are affected by retinopathy and ∼50% of patients with T2D experience kidney disease and neuropathy [4, 5]. Numerous studies have shown that obesity is a major risk factor of T2D onset and progression [6]. Up to 90% of T2D patients are in the overweight or obese category [7, 8].

A growing number of genome wide association studies (GWAS) on diabetes have identified hundreds of T2D-associated loci [9]. A meta-analysis of 32 GWAS comprising of 74,124 T2D cases of European ancestry identified 243 new candidate loci associated with T2D [10]. Another study on 62,892 cases of T2D of European ancestry led to the identification of 143 genetic variants in multiple loci associated with T2D [11], while a multiethnic GWAS meta-analysis covering over 1.4 million participants of European, African American, Hispanic, South Asian and East Asian ancestries led to the detection of 568 T2D associations, of which 318 were identified as novel risk loci [12]. Also, studies of East Asian populations led to the identification of ∼ 90 novel loci associated with T2D [13, 14] and the effect sizes were strongly correlated to Europeans [13, 14].

GWAS have reported several genes that are associated with pancreatic islet β-cell function, insulin sensitivity and glucose metabolism [15]. For instance, *TCF7L2*, *KCNQ1*, *WFS1*, *HNF1B*, *SLC2A2*, *SLC30A8*, *CDKAL1*, *CDKN2A*, *CDKN2B*, *GCK*, *MTNR1B*, and *GIPR* are all associated with pancreatic islet β-cell dysfunction [9]. The transcription factor 7-like 2 (*TCF7L2*), discovered in 2006, was the first locus to be extensively reported in GWAS and is the strongest genetic risk factor associated with T2D to date. Remarkably, *TCF7L2* genetic variants have been replicated in different populations across European, Chinese, Caucasian, South Asian and African ancestries [11, 14, 16–18].

The majority of GWAS are based primarily on European and East Asian populations, while Middle Eastern populations remain largely under-studied. Investigations on replicating known T2D-risk loci in the MENA region have been previously conducted, including in Qatar [19] and Saudi Arabia [20, 21], while only select few GWAS on T2D have been conducted in the region including in Lebanon [22], Circassian and Chechen populations in Jordan [23] and an extended Arab family in the United Arab Emirates [24], with modest sample sizes ranging from 66 to 1384 individuals. GWAS conducted in Lebanon reported only two T2D-associated loci; *CDKAL1* and *TCF7L2* [22], while studies performed in Qatar also replicated only 2 variants in *TCF7L2* [19] and several T2D-associated loci were replicated in Saudi Arabia [20, 21]. Overall, a methodical GWAS on T2D in the MENA region is warranted due to the high and increasing T2D prevalence, specifically in Qatar, where obesity is recognized as the major driver of T2D burden [25].

In this study, we performed the largest and most comprehensive GWAS of T2D in a Middle Eastern population based on whole genome sequencing (WGS) of over 11,000 subjects from the population-based Qatar Biobank (QBB) cohort that included 2765 T2D cases and 8671 controls. We also performed a transethnic meta-analysis of our cohort with GWAS from multi-ancestry individuals (180,834 T2D cases and 1,159,055 controls) [26] and assessed the performance of polygenic risk scoring panels derived from European and multi-ancestries, in the Qatari population. Our findings reveal genetic risk loci associated with T2D, which can be explored further to investigate their roles in T2D onset and progression.

## Methods

### Study participants

Study participants were recruited from the Qatar Biobank (QBB). QBB collects information from native Qatari population and long-term residents (≥ 15 years) [7, 27]. The study cohort consisted of 14,409 participants (aged 18 to 89 years). All participants provided written informed consent prior to participation. This study was approved by the institutional review boards of QBB (E-2019-QF-QBB-RES-ACC-0179-0104) and Hamad Bin Khalifa University, Doha, Qatar (Approval no. 2021-3-78). All participants completed an approved and standardized questionnaire reporting past medical history, lifestyle, diet and physical activity [27]. Medical examination, physical measurements and collection of biological samples (including blood, urine, and saliva) were also conducted.

### Phenotypic data, biochemical measures and patient classification

Participants were determined to have diabetes based on self-reported diabetes status and self-reported use of diabetes medications. We also included those with newly diagnosed diabetes, based on measured HbA1c levels (HbA1c ≥ 6.5%). All biochemical assays were performed at the clinical laboratory of Hamad Medical Corporation, accredited by the College of American Pathologists (CAP). Sandwich electrochemiluminescence immunoassay Elecsys C-Peptide kit (Roche, Basel, Switzerland) was used to measure serum C-peptide levels, while Turbidimetric inhibition immunoassay (Tina-quant HbA1c Gen. 3 kit; Roche) was used to measure serum HbA1c. Enzymatic reference method with hexokinase was used to measure random glucose levels in serum using the COBAS instrument (Roche, Basel, Switzerland).

The study participants were classified into diabetes subtypes based on phenotypic data provided by QBB. First, we removed those with incomplete phenotype data (*n* = 30). Participants with T1DM (*n* = 71) were identified based on self-declared diabetes status, receiving insulin treatment exclusively and with undetectable serum C-peptide levels (< 0.5ng/ml). Participants with T2DM (*n* = 2765) were identified based on self-declared diabetes status, self-reported diabetes medication, or with HbA1c ≥ 6.5%. Those without diabetes and with HbA1c values between 5.7 and 6.4 were classified as prediabetes (*n* = 2271). The remaining participants (*n* = 8671) were considered as diabetes free and were used in the analysis as population-based controls, without removing those with a family history of diabetes.

### Whole-genome sequencing (WGS)

The Qatar Genome project (QGP) provided the WGS data of QBB study participants [28]. A description of WGS and quality control measures has been described previously [29]. Briefly, genomic DNA was extracted from peripheral blood using Qiagen MIDI kit (Qiagen, Germany), following the manufacturer’s protocol and using the automated QIASymphony SP instrument (Qiagen). The DNA integrity was assessed using Caliper Labchip GXII (Perkin Elmer, USA) Genomic DNA assay and was quantified using the Quant-iT dsDNA Assay (Invitrogen, USA). Using the Illumina TruSeq DNA Nano kit (Illumina, San Diego, CA, USA), whole-genome libraries were prepared. Sequencing genomic libraries was performed using HiSeq X Ten (Illumina) for a minimum average coverage of 30X at Sidra Clinical Genomics Laboratory Sequencing Facility (Sidra Medicine, Doha, Qatar). Quality control was conducted using FastQC (v0.11.2) on the generated files and reads were aligned to GRCh38 reference genome. Mapped reads were quality-controlled with Picard (v1.117). For all study participants, a combined variant call file (gVCF) was created containing all genetic variations identified in the QBB study participants.

### Genotyping and quality control

WGS from 14,409 QBB participants was performed by the QGP as previously described [29]. PLINK-v2.0 [30] and Hail 0.2 [31] were utilized for sample-level and variant-level quality controls on the multisample variant call format (VCF) file. Variants with minor allele frequency (MAF) < 0.1%, genotype call rate < 90%, mean depth coverage < 10X and Hardy-Weinberg *P*-value < 1 × 10^− 6^ were removed. A total of 21,318,610 autosomal variants remained for the GWAS. Next, we eliminated samples with call rate of less than 95% (*n* = 18), excess heterozygosity (*n* = 34), duplicate samples (*n* = 45), and samples with ambiguity between self-reported gender and genetically determined sex (*n* = 251). Furthermore, we used multidimensional scaling (MDS) by PLINK v2.0 to identify population outliers which resulted in the removal of 253 participants, leaving a total of 13,808 samples for downstream analysis.

### Genome-wide association study

We performed GWAS analysis using a generalized mixed model association test implemented in the Scalable and Accurate Implementation of Generalized mixed model (SAIGE) [32]. SAIGE is efficient in testing the genetic association of large samples with case-control ratio imbalance and sample relatedness using the saddlepoint approximation [32]. To test the association, we adjusted our model for covariates including age, genderand genetic principal components (PC1-PC4), and performed the analysis with and without body mass index (BMI)-adjustment. We applied the standard genome-wide significant association threshold (*P* < 5 × 10^− 8^).

We identified previously reported genetic variants primarily based on data downloaded from the GWAS catalog. A SNP was considered to be novel if no previous reports were published in the GWAS catalog, Phenoscanner [33], or The Human Genetics Amplifier (HuGeAMP) [34] and NCBI databases (accessed on 1-April-2023) with genome wide significant association (*P* < 5 × 10^− 8^) with diabetes. Also, a locus was considered novel if the lead SNP is mapped more than 500 kb from a previously reported locus and is not in linkage disequilibrium (LD) (r^2^ < 0.2) with the lead SNP of the previously reported locus. Regional associations plots were generated using the LocusZoom tool [35] with LD calculated from QGP data using PLINK. While recombination rates were based on the chromosome build GRCh38. The genomic inflation, Quantile-Quantile plots and Manhattan plots were generated using R (ver. 3.4.0).

### Genome-wide association meta-analysis

We performed a meta-analysis of our GWAS findings from the BMI-unadjusted model and summary statistics from a previously published meta-analysis of T2D GWAS (unadjusted for BMI). The study by Mahajan et. al., included 180,834 T2D cases and 1,159,055 controls of multi-ancestry, comprising of 51.15% European, 28.38% East Asian, 8.28% South Asian, 6.62% African and 5.57% Hispanic individuals [26]. Summary statistics of this study were downloaded from the DIAbetes Genetics Replication and Meta-analysis (DIAGRAM) consortium [36]. METAL was used to combine association statistics in a fixed-effect meta-analysis [37].

### Colocalization analysis of genome-wide significant T2D associations

We conducted Bayesian colocalization analysis for novel T2D association variants identified in the meta-analysis. We used COLOC package using R (ver. 3.4.0) to assess colocalization evidence of GWAS and eQTL signals across specific GTex v8 single-tissue gene expression. We used the default posterior probabilities (p1 = 1 × 10^− 4,^ p2 = 1 × 10^− 4^ and p12 = 1 × 10^− 5^). Colocalization was determined based on posterior probabilities PPH_4_ greater than 0.8, which indicates that a same causative variant is responsible for the observed T2D association and eQTL signals.

### Analysis of polygenic risk score

Polygenic scores (PGS): PGS000036 [10] and PGS000804 [38], were assessed in this study. The scoring files derived from European ancestry (PGS000036) and multi- ancestry (PGS000804) GWAS were downloaded from the PGS catalog (http://www.pgscatalog.org). We used the European-derived PGS based on 171,249 risk variants, due to the large sample size used in score development, which included 117,946 individuals of European ancestry [10]. Mahajan et. al., used the pruning and thresholding method by using variants that were in LD (r2 < 0.6) with a significance threshold of *P* < 0.1, which led to the development of a large SNP scoring panel based on 171,249 variants [10]. We also used the multi-ancestry derived PGS devised by Polfus et. al., [38], based on 582 risk variants reported from the largest published multi-ancestry (Europeans, African Americans, Hispanics, South Asians and East Asians) meta-analysis for T2D (228,499 cases and 1,178,783 controls) [12]. Polfus et al., used a thresholding method with the genome wide significant (*P* < 5.0 × 10^− 8^) variants obtained from the multi-ancestry GWAS from 2,814,564 variants [38]. The performance of PGS were assessed on the QBB cohort using the–score function in PLINK ver. 2.0 [30]. Briefly, the PGS were computed by calculating the sum of risk alleles associated with a trait, weighted by the risk allele effect size from polygenic risk score panels. A total of 469 risk variants from European-derived and 149,851 risk variants from the multi-ancestry derived polygenic panels were identified in our cohort. In concordance with Mahajan et. al., [10], and Polfus et. al., [38], we evaluated the predictive performance of PGS000036 and PGS000804 on the QBB cohort by fitting a logistic regression model, with PGS sum and adjusting for covariates including, gender, age, and principal components (PC1-PC4), with and without BMI. The performance assessment was also based on determining the area under the receiver operating characteristic curve (AUC) for each model and was calculated using R (ver. 3.4.0) to determine whether the two PGS are translatable in the QBB cohort.

## Results

### Study overview

This study was based on WGS and phenotypic data of 8671 controls and 2765 T2D cases. The clinical characteristics of the study cohort are listed in Table 1. The mean age of the control participants was younger (35 ± 10.7 years), compared to cases (51 ± 11.8 years). Altogether, the majority of T2D cases were classified within the overweight (30.6%) or obese (60.8%) categories and presented with a strong family history of diabetes.

**Table 1:** Clinical characteristics of QBB study cohort for GWAS

	Control	Cases
Number of participants (n; %)^♦^	8671 (75.8%)	2765 (24.3%)
Age (y) (mean ± SD)	35 ± 10.69	51 ± 11.8*
Male (n; %)	3819 (44%)	1156 (42%)
Female (n; %)	4852 (56%)	1609 (58%)
BMI (kg/m²)	28.89 ± 5.97	32.22 ± 5.92*
BMI Category: **		
Underweight	239 (2.8%)	3 (0.1%)
Normal weight	2348 (27.1%)	225 (8.1%)*
Overweight	3199 (36.9%)	846 (30.6%)*
Obese	2879 (33.20%)	1681 (60.8%)*
N/A	6 (0.1%)	10 (0.4%)
HbA1c (%)	5.18 ± 0.29	7.39 ± 1.78*
C-peptide (ng/ml)	2.23 ± 1.33	2.85 ± 1.68*
Family History of Diabetes:		
Father (n; %)	3688 (42.5%)	1291 (46.7%)*
Mother (n; %)	3741 (43.1%)	1711 (61.9%)*
Both parents (n; %)	1845 (21.3%)	915 (33.1%)*

^¨^Study cohort comprised of 11,436 participants. Quantitative variables are presented as mean ± standard deviation, qualitative variables are presented as number (% of total in each group), *Statistically significant (*P* < 0.001) compared to non-diabetes controls. BMI: body mass index. **BMI categories were as follows: underweight (< 18.5 kg/m^2^) normal weight (18.5 to 24.9 kg/m^2^), overweight (25 to 29.9 kg/m^2^) and obesity (≥ 30 kg/m^2^)

**Fig. 1:**
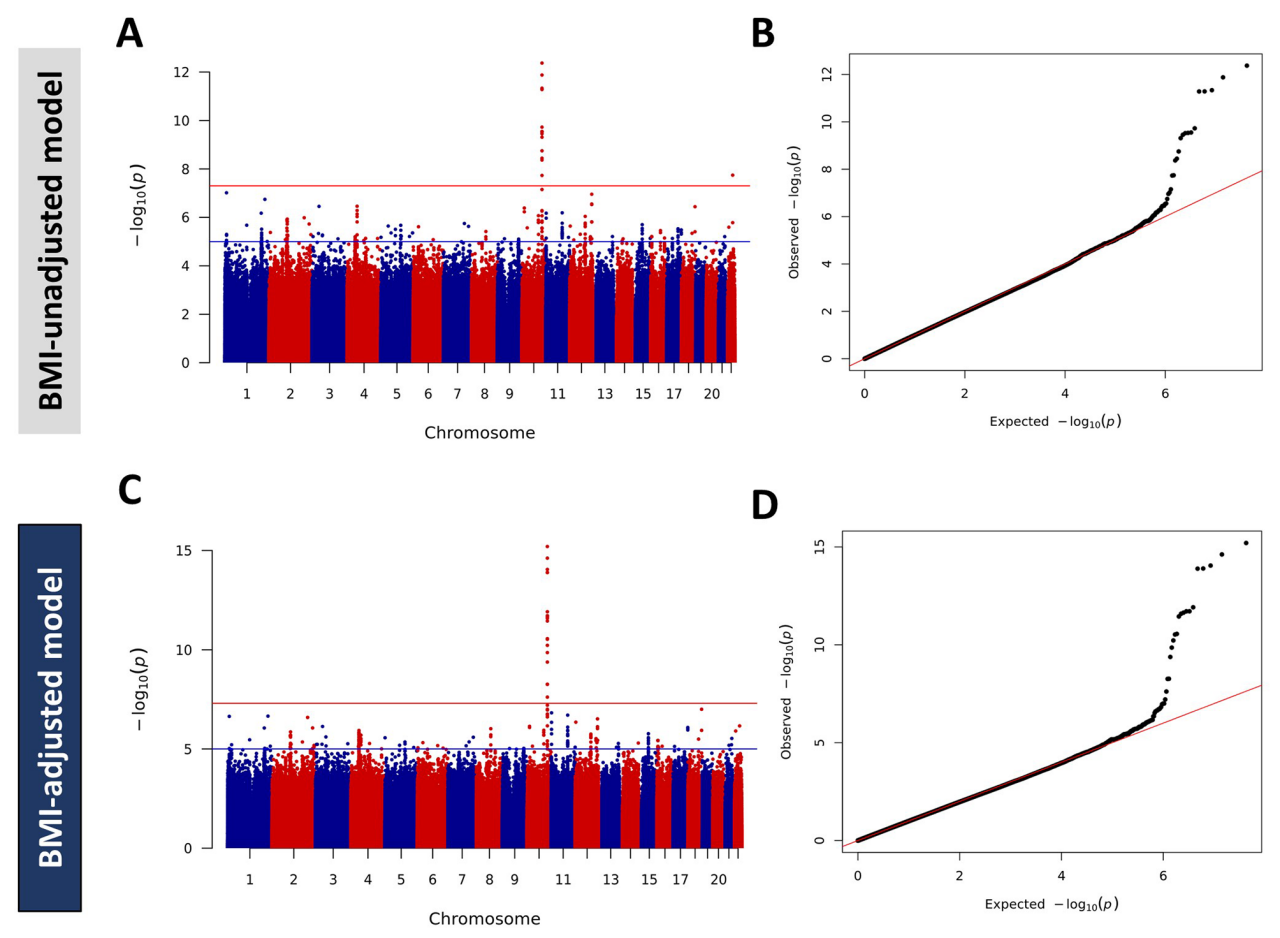
Manhattan Plots and Q-Q Plots for T2D BMI-unadjusted and BMI-adjusted Models. Manhattan plot (**A**, **C**) and Q-Q plot (**B**, **D**) for BMI-adjusted (**A**, **B**) and for BMI-unadjusted (**C**, **D**) GWAS. Manhattan plots represent SNPs (dots) plotted on x-axis in accordance with chromosome position against their corresponding -log10(*P*). The red horizontal line represents the threshold for genome-wide significance (*P* < 5 × 10^− 08^). Q-Q plots represent the quantile distribution of observed *P* values versus the quantile distribution of expected *P* values for all SNPs

Association testing was performed using a mixed model correcting for gender, age, relatedness, and population structure (genetic principal components PC1-PC4) with or without BMI correction, due to the association between T2D and obesity. The Manhattan plots and Q-Q plots of GWAS for the BMI-unadjusted and BMI-adjusted models are illustrated in Fig. 1. The Manhattan plot displayed multiple genome-wide significant loci on chromosome 10 and chromosome 22 in the BMI-unadjusted model (Fig. 1A), and only one locus on chromosome 10 for the BMI-adjusted model (Fig. 1C). Adjusting for covariates with or without BMI resulted in a genomic inflation factor λ_GC_ value of around 1 in both models, suggesting no evidence of genomic inflation (Fig. 1B & D).

### Analysis of previously reported loci associated with T2D in the QBB study cohort

We replicated 220 SNPs (from 93 loci) in the BMI-unadjusted model and 244 SNPs (from 96 loci) in the BMI-adjusted model (*P* < 0.05) that have been previously associated with T2D at genome-wide significance (Supplementary Tables 1 & 2). 203 SNPs were common to both models. 15 SNPs showed genome-wide significance (*P* < 5 × 10^− 8^) in BMI-unadjusted model, while 19 SNPs showed genome-wide significance in BMI-adjusted model, all located at the *TCF7L2* locus. Moreover, 4 SNPs were solely replicated in the BMI-adjusted model. These SNPs showed strong LD (r^2^ > 0.7) with the highest-ranking SNP in *TCF7L2*; rs7903146 (BMI-unadjusted model *P* = 4.26 × 10^− 13^ and BMI-adjusted model *P* = 6.27 × 10^− 16^).

**Fig. 2:**
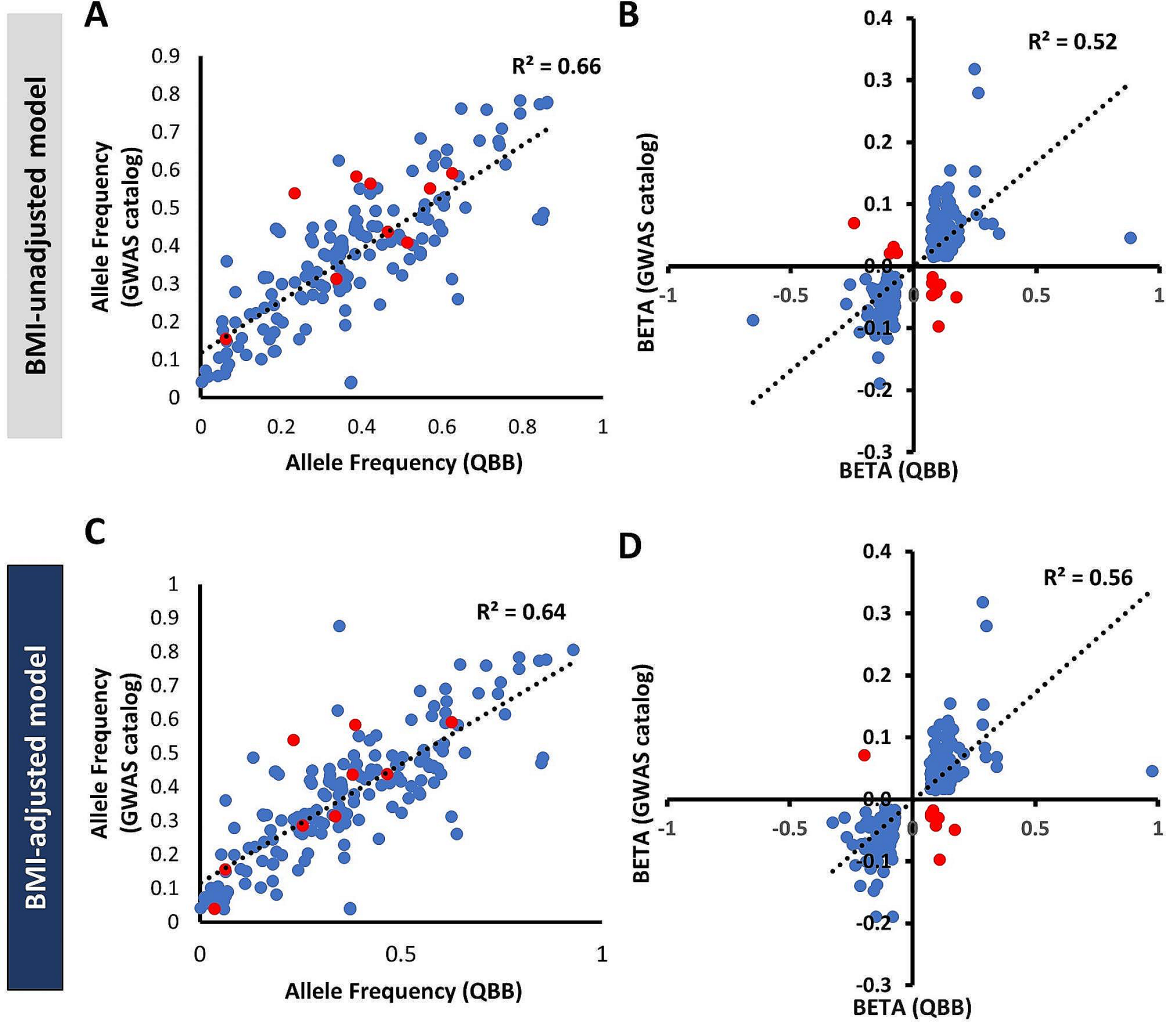
Comparison of Allele Frequencies and Effect sizes (BETA) of Replicated SNPs Identified in the GWAS Catalog and QBB Cohort. Correlation of allele frequencies for replicated loci between QBB and GWAS catalog; **A-B.** BMI-unadjusted and **C-D.** BMI-adjusted models. Blue dots represent SNPs with similar direction of effect size, while red dots represent SNPs with opposite direction of effect size

Next, we compared the allele frequencies (AF) and effect sizes (BETA) of replicated SNPs in our data to those previously reported in the GWAS catalog (Fig. 2). We observed moderate correlation of allele frequencies (BMI-unadjusted, R^2^ = 0.66; BMI-adjusted, R^2^ = 0.64) with those reported in the GWAS catalog. The majority of the identified variants showed consistent direction of effect (Fig. 2B & D) with good correlations to those reported in the GWAS catalog (BMI-unadjusted, R^2^ = 0.52; BMI-adjusted, R^2^ = 0.56). However, 12 SNPs from the BMI-unadjusted model and 10 SNPs from the BMI-adjusted model showed opposite effects but their association *p* values in our data were weak (*P =* 0.01 to 0.05). Moreover, 7 SNPs with opposite effect size overlapped in both models including rs2206734, rs6857, rs7841082, rs3132524, rs1872635, rs4384608 and rs6556925.

Genetic architectures amongst ethnic groups have diverse differences in allele frequencies and disease susceptibility [39]. Subsequently, we assessed the differences in allele frequency of replicated variants across populations (European, African, and South Asian), in relation to the QBB cohort (Supplementary Fig. 1). We observed a high correlation between QBB cohort and European (R^2^ = 0.89), followed by South Asian (R^2^ = 0.79) populations and moderate correlation with the African population (R^2^ = 0.48) for variants replicated in the BMI-unadjusted model. Similarly, QBB cohort and European (R^2^ = 0.87) and South Asian (R^2^ = 0.78) populations showed high correlation, while the African population (R^2^ = 0.47) showed moderate correlation for variants replicated in the BMI-adjusted model (Supplementary Fig. 1).

### Identification of a novel locus associated with T2D

**Fig. 3:**
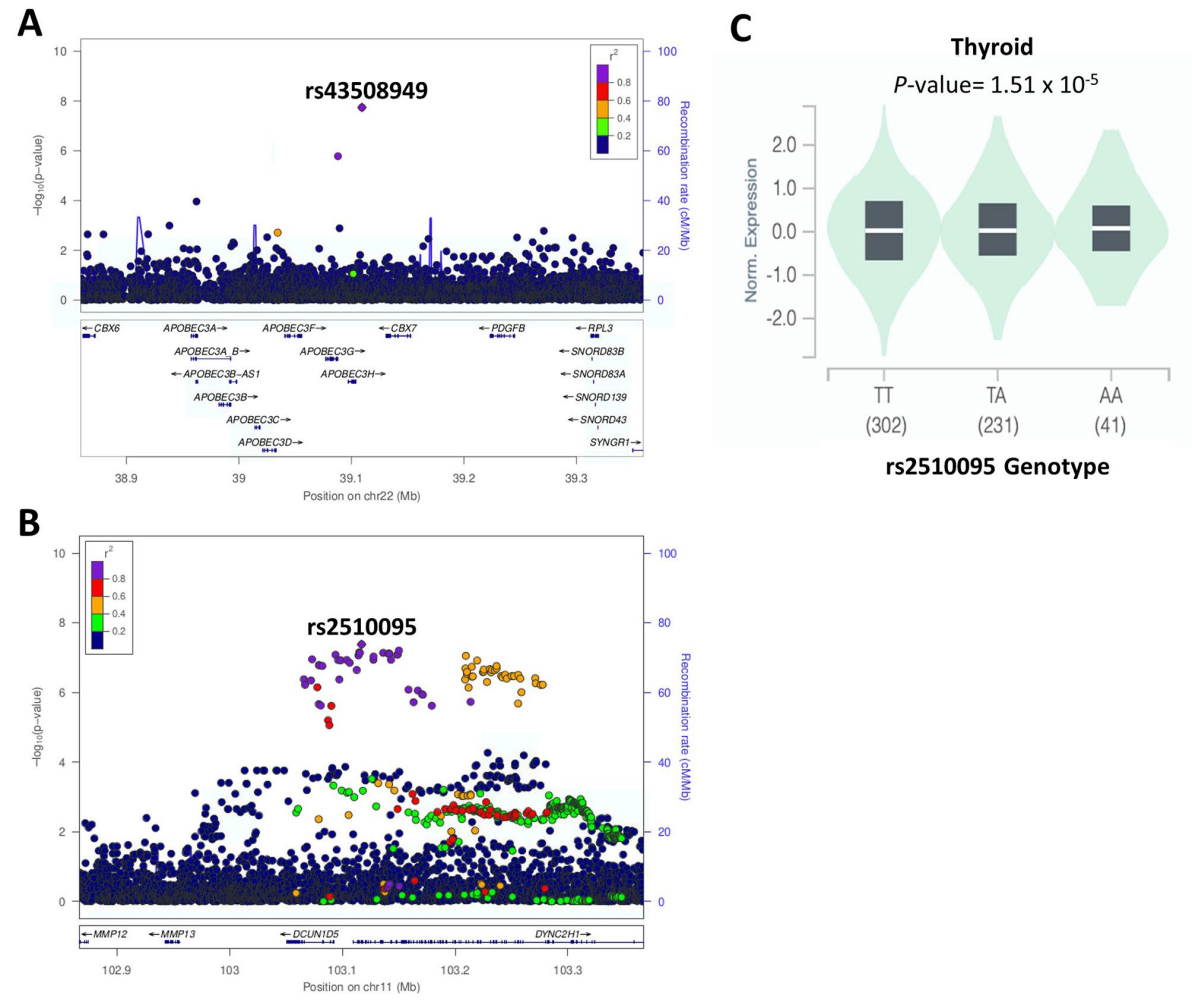
Regional Association Plots of Novel Loci showing Genome-wide Significant Association with T2D. The plot shows the chromosomal position of (**A**) rs143508949 and (**B**) rs2510095 plotted against -log_10_*P* values (based on NCBI human genome build 38). The novel SNP (diamond) and other detected SNPs (circle) are color coded based on LD (square correlation (r2)). (**C**) The lead SNP rs2510095 is a significant eQTL for DYNC2H1 in Thyroid tissues. Box and whiskers plot shows DYNC2H1expression by rs2510095 genotypes

We identified a novel locus in the BMI-unadjusted analysis model tagged by rs143508949, which showed genome-wide significance (*P* = 1.81 × 10^− 8^; BETA = 0.650), on chromosome 22q13.1; located between Apolipoprotein B mRNA editing enzyme catalytic subunit 3 H (*APOBEC3H)* and Chromobox 7 (*CBX7*) genes (Fig. 3A). This SNP was also associated with T2D in the BMI-adjusted model but it did not reach genome wide significance (*P* = 6.8 × 10^− 7^). The identified novel susceptibility locus existed outside of regions previously associated with T2D risk, while no significant variant-trait associations have been reported for rs143508949 in PhenoScanner or NCBI database [33]. The allele frequency of rs143508949 in the QGP cohort was substantially higher (0.022) compared to African (0.0015) and South Asian populations (0.001). In contrast, the AF of rs143508949 was relatively similar to the European population (0.021).

### Identification of novel T2D-associated locus from transethnic meta-analysis of GWAS

To strengthen the power of our study, we performed a transethnic meta-analysis by combining the results of the BMI-unadjusted model from our analysis cohort with summary statistics from the study by Mahajan et. al., which included 80,834 T2D cases and 1,159,055 controls of multi-ancestry populations [26]. We did not perform the analysis on the BMI-adjusted model, since Mahajan et. al., did not adjust for BMI in their overall analysis for the identification of T2D-associated SNPs.

We first investigated the AF and BETA of SNPs that were previously reported at genome-wide significance (*P* < 5 × 10^−8^) in the study by Mahajan et. al., [26] and showed nominal significance (*P* < 0.05) in the QBB cohort (Supplementary Fig. 2). We observed a high correlation of AF with European population (R^2^ = 0.88), followed by South Asian (R^2^ = 0.83) populations and moderate correlation with African population (R^2^ = 0.44). Also, the majority of SNPs showed consistent direction of effects, with a moderate effect size correlation (R^2^ = 0.45). Next, we conducted a meta-analysis which resulted in 19,385 genome-wide significant SNPs (*P* < 5 × 10^− 8^) associated with T2D from the combined dataset (Supplementary Table 3). The highest-ranking SNP in the combined dataset was rs34872471 (*P* = 2.68 × 10^− 528^), located in the *TCF7L2* gene. All genome-wide signals were located in known T2D-susceptibility loci except for one locus located on chromosome 11 (q22.3) within the Dynein, Cytoplasmic 2, Heavy chain 1 (*DYNC2H1)* gene, tagged by rs2510095, which reached genome-wide significance (*P* = 4.18 × 10^− 8^; Beta = 0.0299; effect allele = T; other allele = A) in the transethnic meta-analysis (Fig. 3B) with QBB cohort. The identified novel SNP is not in LD with any previously known signal associated with T2D. Of note, while *DYNC2H1* has not been previously associated with T2D, it has been associated with gestational DM in the PhenoScanner but the association did not reach genome-wide significance. Also, rs2510095 has a strong expression quantitative trait locus (eQTL) for *DYNC2H1* expression in thyroid tissues in the GTEx portal (Fig. 3C), and thyroid dysfunction is associated with increased T2D risk [40].

Next, to assess whether the identified distinct T2D signals are implicated in T2D and tissue-specific gene expression (eQTL), we performed Bayesian colocalization analysis, which showed strong evidence of rs2510095 as a shared eQTL-GWAS signal at the novel locus, *DYNC2H1* identified from the meta-analysis, with a high posterior probability 4 (PP4 = 0.885).

### Assessing the performance of T2D polygenic risk scores in the Qatari cohort

**Fig. 4:**
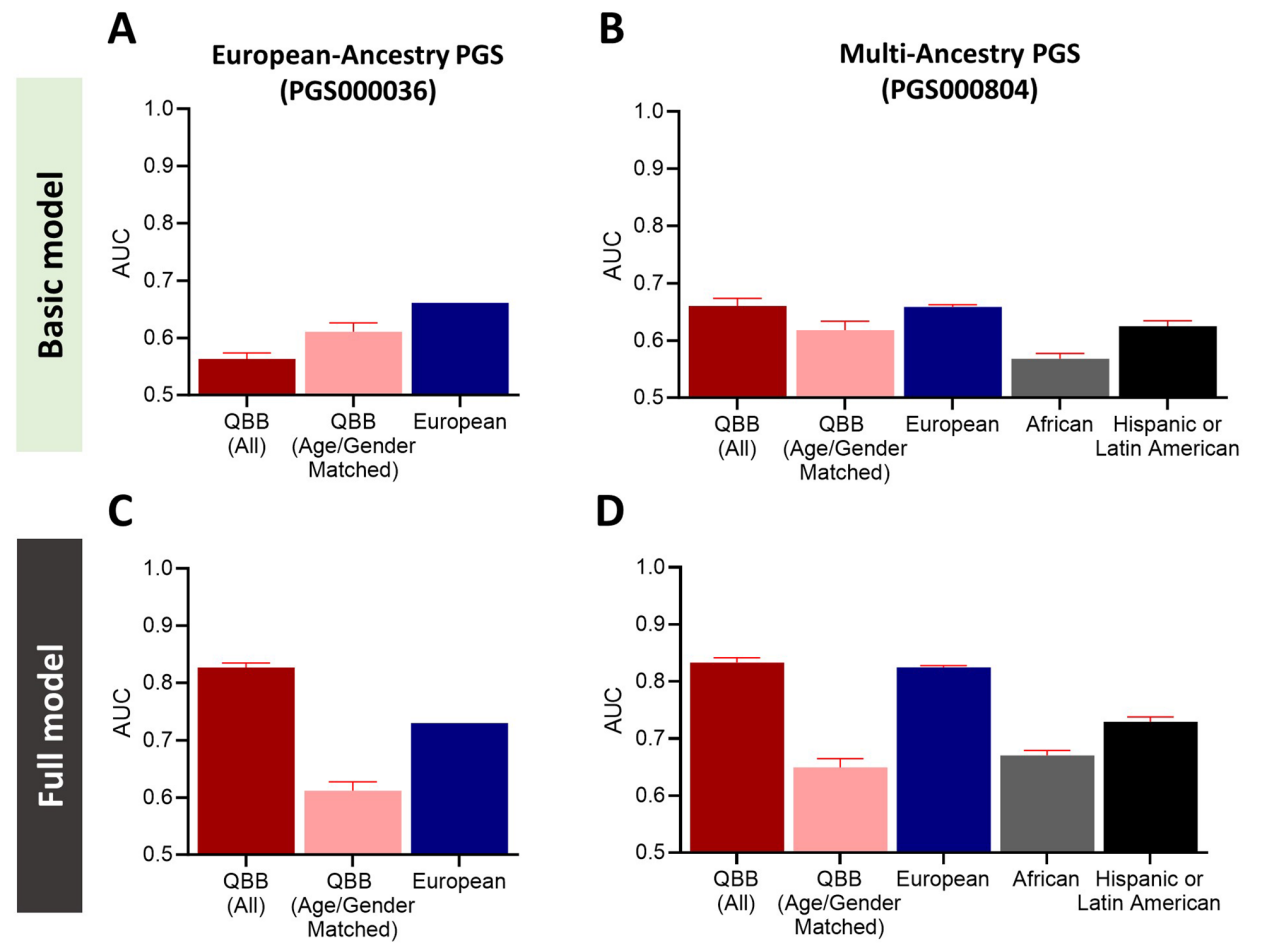
The predictive performances and translatability of T2D polygenic risk scoring panels derived from European- and multi-ancestries. The predictive performances of PGS derived from the European ancestry (PGS000036) and multi-ancestry (PGS000804) when applied to participants from QBB cohort and other ancestries was assessed by the area under the receiver operating curves (AUC) values. (**A**) Bar plots show the predictive performances of the European-ancestry PGS when applied to QBB participants and Europeans [35], and (**B**) the predictive performances of multi-ancestry PGS when applied to participants from QBB, European, African and Hispanic or Latin American ancestries [32] in the basic model (without covariate adjustment). (**C**) Bar plots show the predictive performances of the European-ancestry PGS when applied to QBB and European [34] ancestries and (**D**) the predictive performances of multi-ancestry PGS when applied to participants from QBB, European, African and Hispanic or Latin American ancestries in the full model (with covariate adjustment). Error bars represent 95% confidence interval for AUC. *Predictive performance of the two PGS showed statistical significance (*P* < 0.05) in all tested conditions

We evaluated the predictive performance of applying two PGS panels derived from GWAS of European and multi-ancestry populations on the QBB cohort. We used the developed scoring data accessible in the Polygenic Score Catalog (http://www.pgscatalog.org) [10]. We first evaluated the predictive performances of the PGS panels derived from the European- and multi- ancestry populations by assessing the Area Under the Receiver Operating characteristic curve (AUC). For this analysis, we included all control participants (8671 non-diabetes and 2271 prediabetes) to allow comparison with published data from other ancestries [38, 41, 42]. The predictive performance of the European-ancestry PGS when applied to QBB cohort was low, yielding an AUC of 0.56 compared to its performance when applied to participants of European (AUC = 0.66; Fig. 4A). Notably, inclusion of covariates such as age, gender and BMI greatly improved the predictive performance of European-ancestry PGS model when applied to QBB (AUC = 0.83), which was even higher than its application on Europeans (AUC = 0.73, Fig. 4C). However, this is mostly explained by the significant age differences between cases and controls in the QBB cohort (Table 1). Next, we selected a random set of sex and age-matched cases and controls from our QBB cohort (Supplementary Table 4) and repeated the PGS analysis which resulted in a lower performance (AUC = 0.61) compared to that reported for European ancestry (AUC = 0.73).

Similarly, the Multi-ancestry PGS showed moderate predictive performance on QBB cohort (AUC = 0.66; Fig. 4B), which was improved by the inclusion of covariates (AUC = 0.83; Fig. 4D). Moreover, a similar trend was reported when the Multi-ancestry PGS was applied on Europeans (AUC = 0.66 improved to AUC = 0.83 with inclusion of covariates [38, 41, 42]. Assessing the predictive performance of Multi-ancestry PGS on the age-matched cohort also resulted in lower performance (AUC = 0.65) compared to that previously reported for European ancestry (AUC = 0.83) but higher than the European PGS applied to the age matched cohort (AUC = 0.61).

## Discussion

Over 700 T2D risk loci have been identified in recent years in studies predominantly conducted on European, Asian and African populations [43]. However, studies on the genetic basis of T2D are under-investigated in the Middle East, despite the rapidly increasing disease prevalence. To our knowledge, this is the first population-based GWAS conducted for T2D in Qatar and in the wider Middle Eastern region.

WGS enables complete coverage of the human genome and facilitates the identification of loci and strong functional candidate genes associated with a trait. Comparison of the allele frequencies and effect sizes of the replicated genetic variants to the GWAS catalog and 1000 Genome Project populations (African, European, and South Asian) showed high similarity in effect-size distribution for the majority of the replicated genetic variants.

We detected several loci that have been previously associated with T2D, including variants in *TCF7L2,* which has a strong genetic association with T2D. We also identified a genome-wide significant locus tagged by rs143508949 on chromosome 22 (q13.1), located between *APOBEC3H* and *CBX7* genes in the BMI-unadjusted model. rs143508949 was not statistically significant in the study by Mahajan et. al., [26], however a nearby SNP located 123 kb away showed association with T2D in the Mahajan Study but at a less stringent *P* value (rs2076109; *P* = 6.5 × 10^− 4^). *APOBEC3H* is a member of the Apolipoprotein B mRNA Editing Catalytic Polypeptide-like (APOBEC) family, which has been previously reported to have a significant impact on the instability of cancer genome [44]. Moreover, APOBEC3 proteins can inhibit retroelements (Res) such as *Alu.* Hypomethylation levels of *Alu* are associated with high fasting blood sugar, HbA1c and blood pressure in leukocytes of T2D patients [45]. In addition, *CBX7* is a component of Polycomb group (PcG) of proteins, which form Polycomb Repressive complexes (PRCs) to regulate gene expression through histone modifications [46]. PcG proteins play important roles in pancreatic differentiation, homeostasis and β-cells maturation. Studies have shown that the dysregulation of epigenetic mechanisms mediated by PRCs is a hallmark of β-cell failure in diabetes. Moreover, the dysregulation of PRC contributes to transcriptional changes associated with β-cell dysfunction in T2D [47, 48]. These reports suggest that variants in these genes could impact T2D development and progression. Moreover, while a SNP between *APOBEC3H* and *CBX7* was previously associated with coronary artery calcified atherosclerotic plaque in diabetes subjects of African American ancestry, it did not show genome-wide significance [49]. Overall, validation in a replication cohort is required to ascertain the genome-wide association between our identified SNP and T2D.

The transethnic meta-analysis led to the identification of a novel SNP, rs2510095 in a novel locus in *DYNC2H1* gene that reached genome-wide significance. To our knowledge *DYNC2H1* has not previously been associated with T2D but has been associated with lipid storage and other syndromes including Jeune syndrome and Short rib-polydactyly syndrome [50–52]. Knock-down of *DYNC2H1* increased lipid accumulation in adipocytes [52], while the protein encoded by *DYNC2H1* is a component of the cytoplasmic dynein-2 complex in cilia and plays a vital role in cilia biogenesis and signal transduction [53]. Inhibition of cilia motility impaired Calcium (Ca^2+^) influx and insulin secretion [54] and cilia-related genes have been shown to be dysregulated in T2D patients and associated with obesity [55]. The lead SNP from this locus also has a strong eQTL for DYNC2H1 expression in thyroid tissue. Our findings highlight the association between a novel variant in *DYNC2H1* with T2D, which may be explored further.

The PGS panel derived from multi-ancestry GWAS showed higher predictability and translatability than European ancestry-based GWAS, when implemented to the QBB cohort. The performance metrics of the European ancestry PGS panel for T2D, when applied to our cohort showed lower predictive performance (AUC = 0.57) compared to its application on European Cohorts (AUC = 0.66) [41]. These results are in accordance with a previously published study based on QBB cohort for several other clinically relevant traits [29]. Interestingly, our analyses showed that the performance of European PGS was higher in QBB cohort (AUC = 0.83) when age, sex, and BMI were included in the model compared to European cohort (AUC = 0.73) [41]. This is mostly explained by the differences in age between cases and non-diabetes controls in our cohort since similar analysis performed on a random subset of age- and sex-matched individuals yielded lower AUC of 0.61. In contrast, multi-ancestry PGS when applied to European cohort showed similar predictive performance (AUC = 0.66) [38], compared to QBB cohort (AUC = 0.66). While, the predictivity increased to (AUC = 0.83) for the European cohort [38] and for QBB cohort (AUC = 0.83) when age, sex and BMI were included in the model but this was mostly driven by age differences between cases and controls in our cohort. The high performance observed following inclusion of covariates is reported by several studies, demonstrating the association of BMI and age with increasing T2D risk [56, 57]. Moreover, studies have also shown that disease prediction in diabetes and cancer is enhanced using multiethnic based PGS panels [58–60]. However, while the multi-ancestry derived PGS performed better in the Qatari population in the sex- and age-matched cohort compared to the European-derived PGS, its performance was still lower compared to that applied to Europeans. These findings highlight the importance of deriving a Qatari-specific PGS with higher predictive performance.

Our study has several limitations. Although this is the largest study in a MENA population, but still relatively small compared to the latest GWAS in European ancestries. Also, our control group was younger than those with T2D, although we have adjusted for age in our GWAS analysis. Moreover, we were unable to derive a Qatari-specific PGS panel due to the lack of a separate cohort to evaluate its performance. Overall, despite these challenges the identified novel loci and genetic variants highlighted novel potential targets for T2D etiology in Qatar, which may be explored further in functional studies to determine the molecular pathways affected by them.

## Conclusion

We conducted the largest GWAS of T2D in the MENA region and replicated many previously reported loci and identified a novel susceptibility locus on chromosome 22. The majority of replicated loci showed consistent direction of effect and high allele frequency correlation compared to previous reports. Our trans-ethnic GWAS meta-analysis identified one additional novel susceptibility locus tagged by a SNP located in *DYNC2H1,* which is also associated with expression in tissues relevant to insulin resistance. Assessing PGS derived from European- and multi-ancestries in the Qatari population showed higher predictive performance of the multi-ancestry panel compared to the European panel. Our study provides new insights into the genetic architecture of T2D in the Middle Eastern population of Qatar.

### Electronic supplementary material

Below is the link to the electronic supplementary material.

Supplementary Figures

Supplementary Table 1

Supplementary Table 2

Supplementary Table 3

Supplementary Table 4

## Data Availability

The data analyzed in this study are subject to the following licenses/restrictions: the raw whole-genome sequence data from Qatar Biobank are protected and are not available for deposition into public databases due to data privacy laws. Access to QBB/QGP phenotype and whole-genome sequence data can be obtained through an ISO-certified protocol, which involves submitting a project request at https://www.qatarbiobank.org.qa/research/how-apply, subject to approval by the Institutional Review Board of the QBB. Requests to access these datasets should be directed to https://www.qatarbiobank.org.qa/research/how-apply.
