# Correction: Genome-wide association study and trans-ethnic meta-analysis identify novel susceptibility loci for type 2 diabetes mellitus

**DOI:** 10.1186/s12920-024-01903-w

**Published:** 2024-05-16

**Authors:** Asma A Elashi, Salman M Toor, Umm-Kulthum Ismail Umlai, Yasser A. Al-Sarraj, Shahrad Taheri, Karsten Suhre, Abdul Badi Abou-Samra, Omar M. E. Albagha

**Affiliations:** 1 College of Health and Life Sciences (CHLS), Foundation (QF), Hamad Bin Khalifa University (HBKU), Education City, DohaP.O. Box 34110, Qatar; 2 Qatar Genome Program (QGP), Qatar Foundation Research, Development and Innovation, Qatar Foundation (QF), DohaP.O. Box 5825, Qatar; 3 Qatar Metabolic Institute, Hamad Medical Corporation, P.O. Box 3050, Doha, Qatar; 4 Bioinformatics Core, Weill Cornell Medicine-Qatar, Education City, DohaP.O. Box 24144, Qatar; 5 Department of Biophysics and Physiology, Weill Cornell Medicine, New York, 510065 USA; 6 Centre for Genomic and Experimental Medicine, Institute of Genetics and Cancer, University of Edinburgh, Edinburgh, EH4 2XU UK

**Correction to: Elashi et al. BMC Medical Genomics (2014) 17:115**

10.1186/s12920-024-01855-1.

Following the publication of the original article, the author notified that there were errors in Supplementary Fig. 1A. Two values need to be corrected to match the significant figures mentioned in the corresponding text in the article, while another value also needs to be corrected.

**Incorrect**.


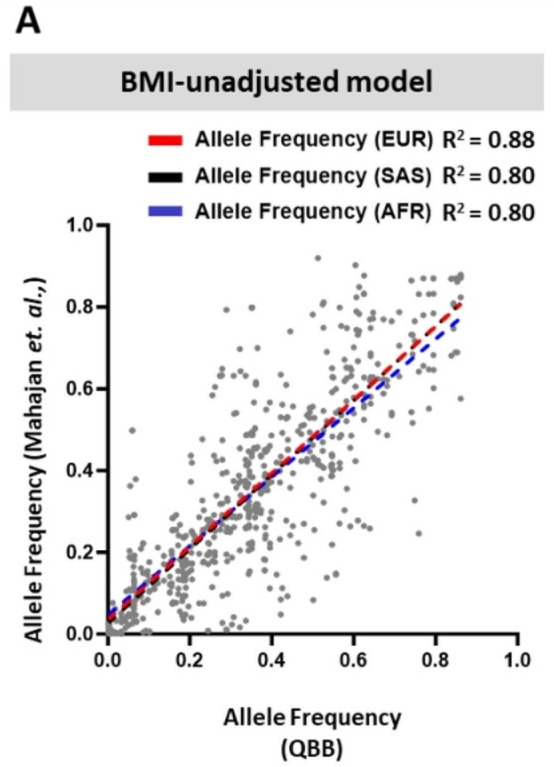


**Correct**.


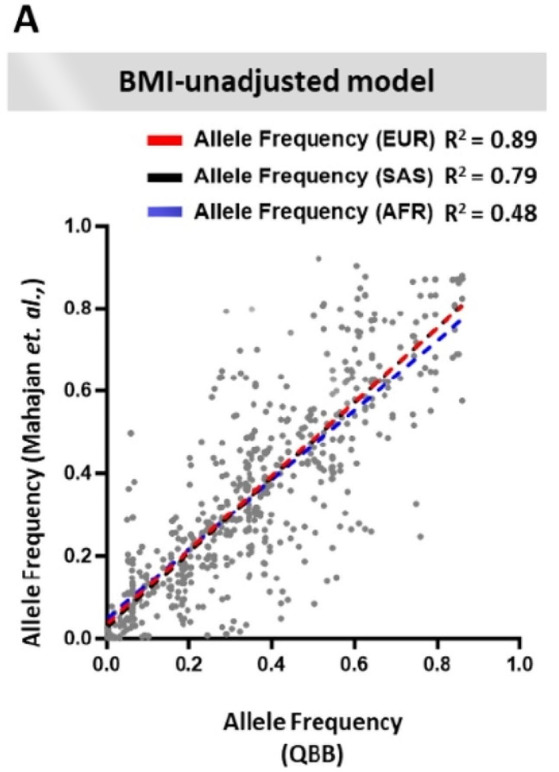


The original article has been corrected.

### Electronic supplementary material

Below is the link to the electronic supplementary material.

Supplementary Material 1

