# Supplementary material for: Genome-wide association study and trans-ethnic meta-analysis identify novel susceptibility loci for type 2 diabetes mellitus
Source: BMC Med Genomics. 2024 Apr 29;17:115. doi: 10.1186/s12920-024-01855-1 (PMC11059680; doi:10.1186/s12920-024-01855-1)
Supplement: Supplementary file 1 — Supplementary Figures [file 12920_2024_1855_MOESM1_ESM.pdf]

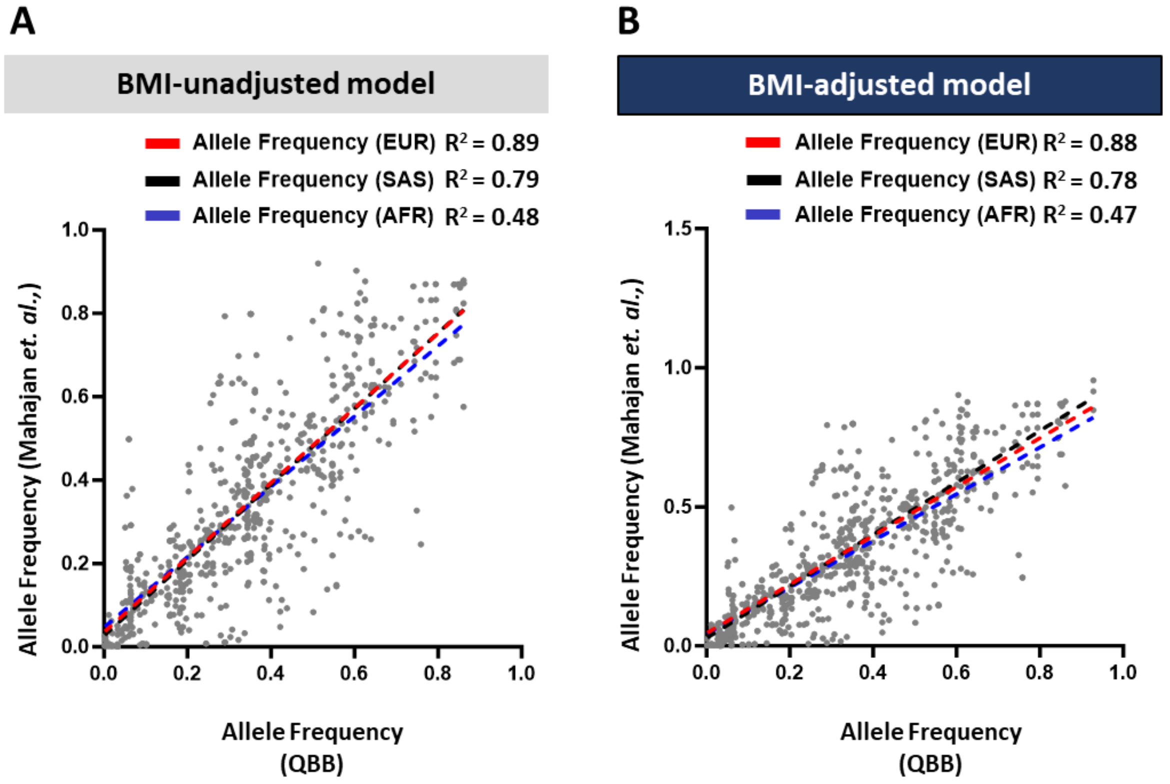


**Supplementary Figure 1. Comparison of Allele Frequencies of replicated SNPs between different populations and QBB Cohort.** Correlation of allele frequencies between Mahajan *et. al.,* (26) and QBB in **A.** BMI-unadjusted and **B.** BMI-adjusted model. EUR: European; AFR: African; SAS: South Asian; QBB: Qatar Biobank.

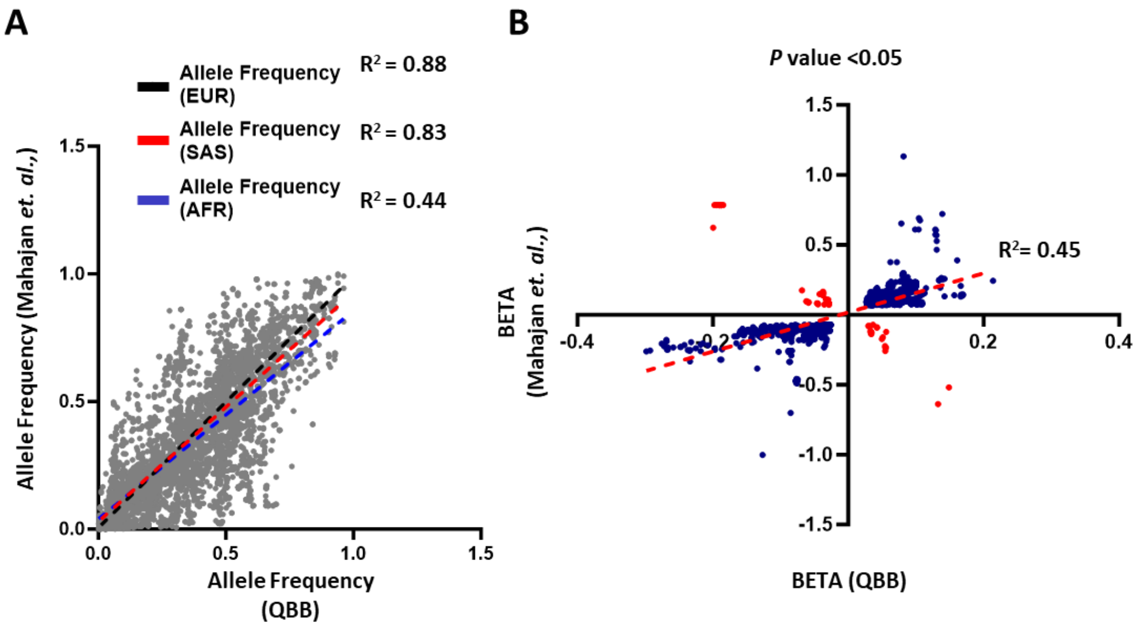


**Supplementary Figure 2. Comparison of Allele Frequencies and BETA of SNPs with genome-wide significance in Mahajan *et. al.,* (26) and nominal significance in QBB cohort. A.** Correlation of allele frequencies between Mahajan *et. al.,* (26) and QBB cohort. **B.** Plot represents BETA of replicated SNPs. Blue dots represent SNPs with similar direction of effect size, while red dots represent SNPs with opposite direction of effect size. EUR: European; AFR: African; SAS: South Asian; QBB: Qatar Biobank.
