# Supplementary material for: Genome-wide association study and trans-ethnic meta-analysis identify novel susceptibility loci for type 2 diabetes mellitus
Source: BMC Med Genomics. 2024 Apr 29;17:115. doi: 10.1186/s12920-024-01855-1 (PMC11059680; doi:10.1186/s12920-024-01855-1)
Supplement: Supplementary file 5 — Supplementary Table 4 [file 12920_2024_1855_MOESM5_ESM.pdf]

Supplementary Table 4. Clinical characteristics of QBB study (Age & Gender Matched) cohort for PGS.

	**Control**	**Cases**
**Number of subjects**¨	2469	2469
**Age (mean ± SD)**	50±11.1	50±11.1
**Male**	1020	1020
**Female**	1449	1449
**BMI (kg/m^2)****	30±6.03	32±5.94*
**Underweight**	29 (1.2%)	2 (0.1%)*
**Normal weight**	374 (15.1%)	199 (8.1%)*
**Overweight**	844 (34.2%)	754 (30.5%)*
**Obese**	1222 (49.5%)	1513 (61.3%)*
**N/A**		-
**HbA1c (%)**	5.46±0.44	7.37±1.79*
**C-peptide (ng/ml)**	2.62±1.56	2.85±1.68*
