# Supplementary material for: Correction: Genome-wide association study and trans-ethnic meta-analysis identify novel susceptibility loci for type 2 diabetes mellitus
Source: BMC Med Genomics. 2024 May 16;17:131. doi: 10.1186/s12920-024-01903-w (PMC11097563; doi:10.1186/s12920-024-01903-w)
Supplement: Supplementary file 1 — Supplementary Material 1 [file 12920_2024_1903_MOESM1_ESM.pdf]

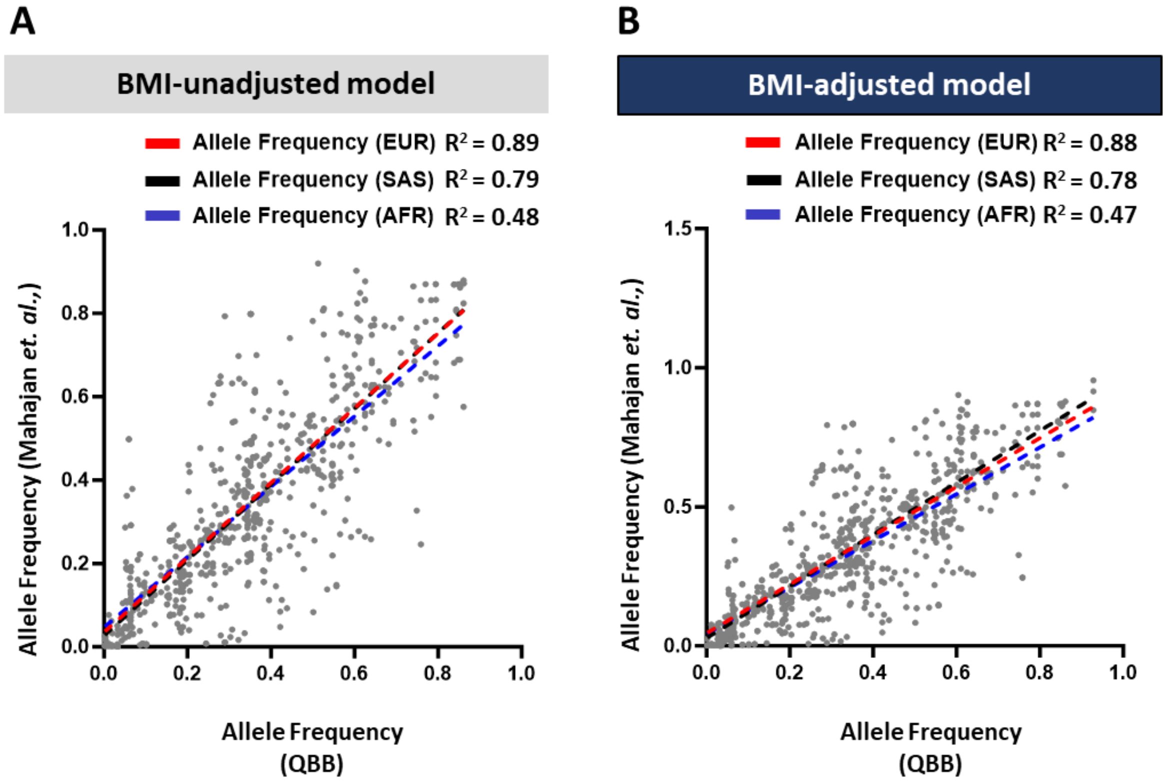

Supplementary Figure 1. Comparison of Allele Frequencies of replicated SNPs between different populations and QBB Cohort. Correlation of allele frequencies between Mahajan *et. al.,* (26) and QBB in **A.** BMI-unadjusted and **B.** BMI-adjusted model. EUR: European; AFR: African; SAS: South Asian; QBB: Qatar Biobank.

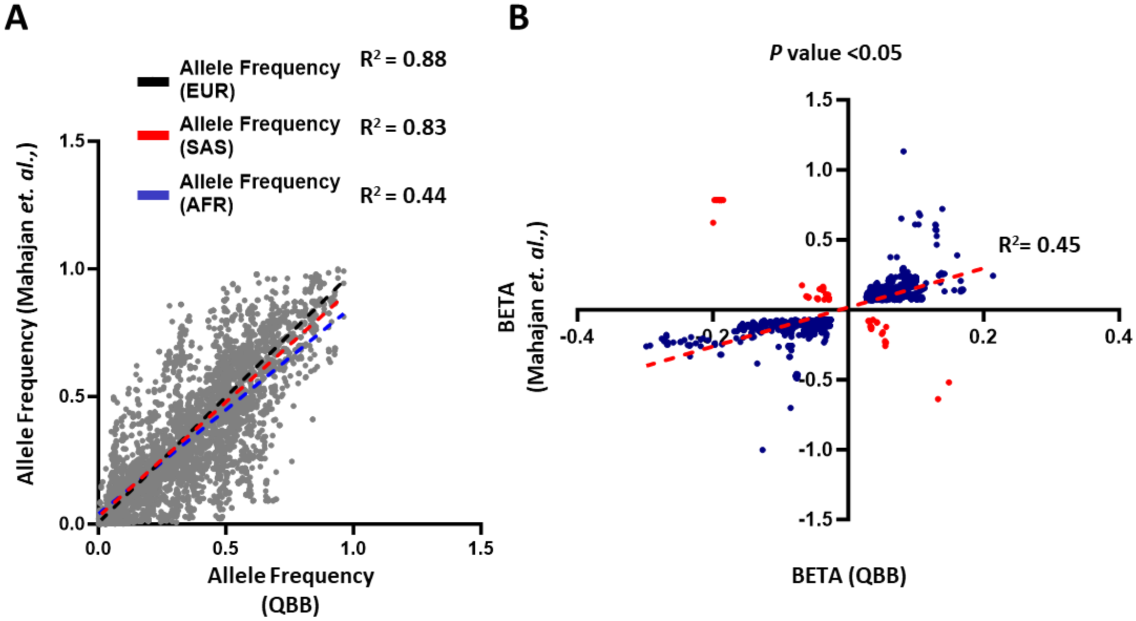

Supplementary Figure 2. Comparison of Allele Frequencies and BETA of SNPs with genome-wide significance in Mahajan *et. al.,* (26) and nominal significance in QBB cohort. A. Correlation of allele frequencies between Mahajan *et. al.,* (26) and QBB cohort. **B.** Plot represents BETA of replicated SNPs. Blue dots represent SNPs with similar direction of effect size, while red dots represent SNPs with opposite direction of effect size. EUR: European; AFR: African; SAS: South Asian; QBB: Qatar Biobank.
